# C-Linked 8-aryl guanine nucleobase adducts: biological outcomes and utility as fluorescent probes

**DOI:** 10.1039/c6sc00053c

**Published:** 2016-02-24

**Authors:** Richard A. Manderville, Stacey D. Wetmore

**Affiliations:** a Department of Chemistry & Toxicology , University of Guelph , Guelph , ON , Canada N1G 2W1 . Email: rmanderv@uoguelph.ca; b Department of Chemistry & Biochemistry , University of Lethbridge , Lethbridge , AB , Canada T1K 3M4 . Email: Stacey.Wetmore@uleth.ca

## Abstract

Aryl radical species derived from enzymatic transformations of aromatic mutagens preferentially react at the 8-site of the guanine (G) nucleobase to afford carbon-linked 8arylG adducts. The resulting lesions possess altered biophysical and genetic coding properties compared to the precursor G nucleoside in B-form DNA. Unlike other adducts, these lesions also possess useful fluorescent properties, since direct attachment of the 8aryl ring extends the purine π-system to afford G mimics with red-shifted excitation maxima and emission that can be sensitive to the microenvironment of the 8arylG base within nucleic acid structures. In B-form DNA, 8arylG adducts are disruptive to duplex formation because they prefer to adopt the *syn*-conformation about the bond connecting the nucleobase to the deoxyribose backbone, which perturbs Watson–Crick (WC) H-bonding with the opposing cytosine (C). Thus, in a B-form duplex, the emissive properties of 8arylG adducts can be employed as a tool to provide insight into adduct conformation, which can be related to their biological outcomes. However, since Gs preferentially adopt the *syn*-conformation in left-handed Z-DNA and antiparallel G-quadruplex (GQ) structures, 8arylG lesions can be inserted into *syn*-G positions without disrupting H-bonding interactions. In fact, 8arylG lesions can serve as ideal fluorescent probes in an antiparallel GQ because their emission is sensitive to GQ folding. This perspective outlines recent developments in the biological implications of 8arylG formation together with their utility as fluorescent G analogs for use in DNA-based diagnostic systems.

## Abstract 2

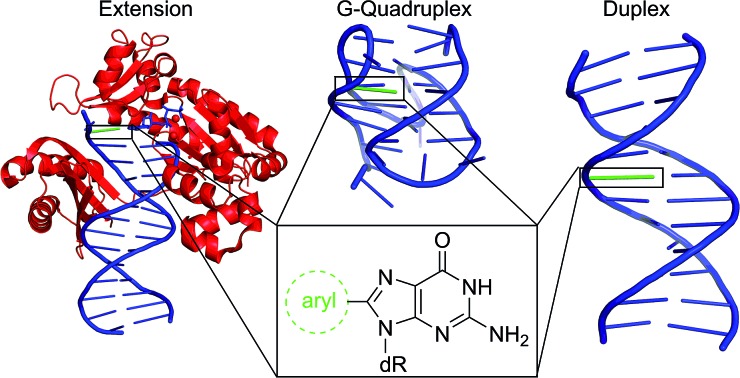
We summarize the utility and biological outcomes resulting from direct attachment of aryl residues to the 8-site of the guanine nucleobase to afford mutagenic lesions and fluorescent probes in G-quadruplex structures.

## Introduction

DNA provides a useful scaffold from which to design novel molecules with applications in diagnostics,^1^ therapeutics,^2^ nanoscience,^3^,^4^ and genomics.^5^ Chemical modification of the DNA nucleobases,^5^–^11^ or the sugar–phosphate backbone,^12^,^13^ can provide new desirable properties that expand the scope of DNA applications. Nucleobase modifications are of particular interest because these can change DNA base-pairing to expand the genetic alphabet,^6^ afford new redox^7^,^8^ or fluorescent^9^–^11^ sensing bases, or affect the gene function in different tissues.^5^

**Fig. 1:**
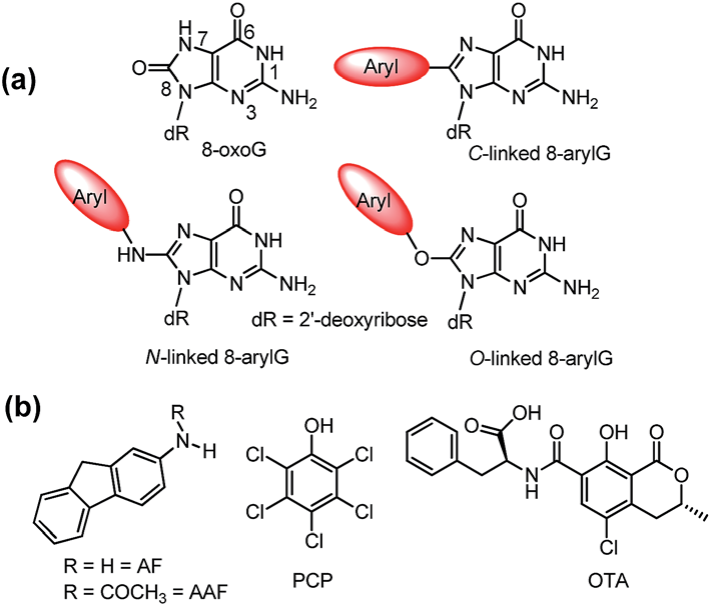
(a) Structures of 8oxoG and 8arylG adducts with different linkages. (b) Structures of mutagens that produce the different 8arylG adducts.

In contrast to the design and synthesis of modified DNA bases for specific applications, useful nucleobase modifications have been identified by studying the cellular implications of DNA damage.^14^ This is particularly true for DNA adducts (addition products), which stem from attack of the human genome by reactive chemical species. For example, 7,8-dihydro-8-oxo-2′-deoxyguanosine (8oxoG, Fig. 1) is a biomarker for oxidative DNA damage.^8^ The biological impact of 8oxoG stems from the 8-keto conformation, which permits 8oxoG to form a stable Hoogsteen base pair with adenine.^15^ Consequently, 8oxoG is mutagenic and unrepaired 8-oxoG lesions cause G → T transversions in cells.^16^ Remarkably, 8oxoG possesses an even lower oxidation potential (*E*_1/2_ = 0.74 V *versus* NHE^17^) than the parent G (*E*_1/2_ = 1.29 V *versus* NHE^18^), permitting its selective oxidation in DNA substrates. Thus, the 8oxoG lesion has become an effective redox tool for studying DNA electron transfer^19^,^20^ and detecting single-base mismatches.^21^

Beyond the formation of 8oxoG, aryl groups can covalently attach to the 8-position of G following enzymatic transformations of different mutagens and carcinogens, and the resulting lesions have been classified as either nitrogen-, carbon- or oxygen-linked 8arylG adducts (Fig. 1).^22^–^28^ Extensive efforts have focused on the biological impact of the N-linked derivatives because these lesions are produced by notorious chemical carcinogens that are present in, for example cigarette smoke, fossil fuels and cooked meats.^29^ The associated adducts have been detected in human cells,^30^,^31^ including lesions arising from 2-aminofluorene (AFG) and *N*2-acetylaminofluorene (AAFG, Fig. 1b), which serve as prototype adducts to elucidate the molecular mechanisms of mutagenesis induced by arylamine carcinogens.^32^–^34^ In contrast to the N-linked variants, relatively little is understood about the structure and biological impact of O- and C-linked 8arylG adducts. Nevertheless, these lesions also arise from our exposure to a variety of sources. For example, O-linked adducts have been connected with pentachlorophenol (PCP, Fig. 1b) found in pesticides, disinfectants and wood preservatives,^22^,^23^,^25^,^27^ while the food mutagen ochratoxin A (OTA) arising from several species of (*Aspergillus* and *Penicillium*) fungi has been linked to the formation of a C-linked adduct (Fig. 1b).^35^–^37^

In terms of structure, the C-linked 8arylG adducts are unique because they lack the flexible tether that separates the aryl component from the G nucleobase in the N- and O-linked lesions. As a result, C-linked aryl moieties extend the purine π-system, which commonly results in fluorescent nucleobase analogues.^9^–^11^ Emissive DNA bases enjoy a wide range of applications that include reporters of nucleic acid structure and function;^38^–^43^ detectors of nucleobase damage, single nucleotide polymorphisms (SNPs) and mismatch dynamics;^44^–^46^ probes for understanding protein–DNA interactions;^47^–^50^ components of aptamers to provide a fluorescent signal upon target binding;^51^–^53^ and oligonucleotide-based therapeutics.^54^

**Fig. 2:**
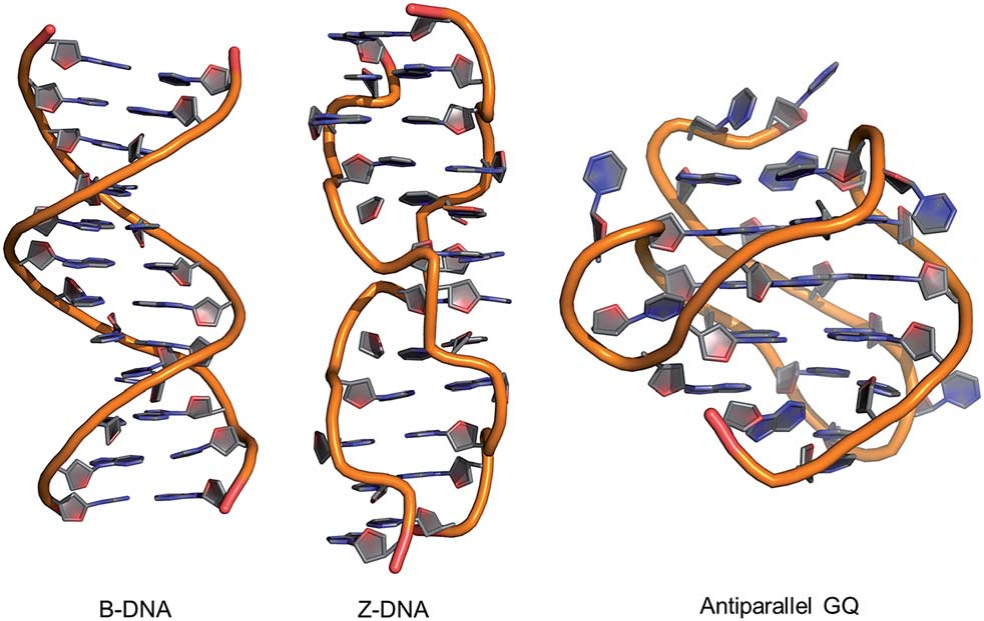
Structures of B-form and Z-form DNA duplexes and an intramolecular antiparallel G-quadruplex (GQ).

Ideal fluorescent probes provide emission switching properties while retaining the native behavior of the nucleic acid system being studied. Indeed, it is considered detrimental for probe incorporation to produce a major perturbation to duplex stability, H-bonding interactions, or folding characteristics. Modifications to the 8-position of G offer an attractive avenue to fluorescent probes because this site is not involved in canonical base-pairing interactions.^38^ Nevertheless, fluorescent C-linked 8arylG adducts are detrimental to the stability of a B-DNA duplex (Fig. 2) because they prefer to adopt the *syn*-conformation.^55^ In this scenario, the emissive properties of 8-arylG adducts can be exploited as a tool to provide insight into adduct conformation within the helix,^24^,^28^ which can be related to biological outcome, as discussed above for 8oxoG.

Alternative oligonucleotide structures, such as Z-DNA^56^,^57^ and antiparallel G-quadruplexes (GQs),^58^,^59^ are also biologically relevant (Fig. 2). For example, Z-DNA plays a role in transcription and has been implicated in mutagenesis,^57^ while GQs are active drug targets.^59^ Both Z-DNA and GQs contain Gs that preferentially adopt the *syn*-conformation. In these DNA structures, 8arylGs can occupy *syn*-G positions without disturbing the overall stability and H-bonding interactions in the nucleic acid. In Z-DNA produced by alternating purine–pyrimidine sequences, the purines adopt the *syn*-conformation, while pyrimidines maintain the *anti*-conformation, leading to a left-handed “zigzag” helix. Antiparallel GQs are composed of stacked G-quartets, which are stabilized by certain cations and contain alternating *syn*- and *anti*-Gs, and intervening sequences, which are extruded as single strand loops. The ability of 8arylG lesions to promote the formation of Z-DNA and GQs may provide a rationale for their biological activity.^60^–^63^ In antiparallel GQs, 8arylGs also behave as ideal fluorescent probes, exhibiting emission that is sensitive to GQ folding.^38^,^42^,^52^,^53^

In this perspective, we discuss the synthesis, properties, biological impact and applications of 8arylG adducts. We start by summarizing the nucleoside structures and properties, and methods for their incorporation into oligonucleotide substrates. The structural and biological impact of 8arylG adducts within the B-DNA “*NarI*” recognition sequence (5′-G_1_G_2_CG_3_CC) is then presented, which highlights a comparison between the properties of the C-linked 8arylG adducts with the established biological properties of the N-linked variants. Novel carcinogenesis mechanisms for C-linked 8arylG adducts are then discussed based on their unique ability to promote Z-DNA and GQ formation. Finally, the utility of fluorescent 8arylG probes in duplex–GQ exchange systems is presented, which provides a signalling platform in biosensors. Together, the work highlighted within points toward a rich future for 8arylG probes in aptasensor development.

## Nucleoside synthesis, structure and properties

**Fig. 3:**
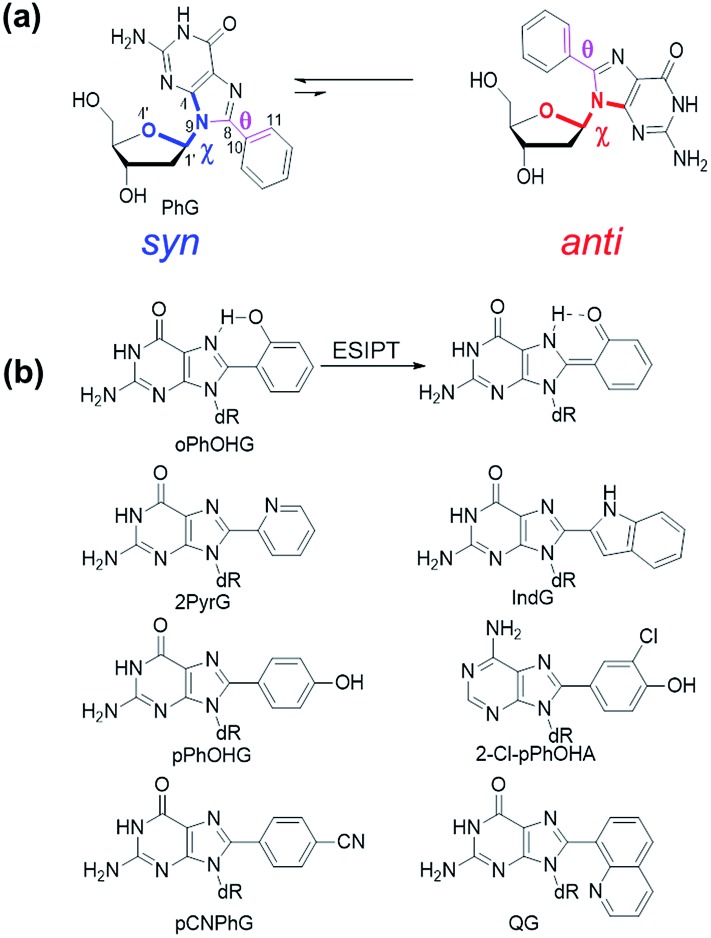
(a) *Syn* (left) and *anti* (right) structures of PhG. The dihedral angle *χ* (∠(O4′–C1′–N9–C4)) defines the glycosidic bond orientation to be *anti* (*χ* = 180 ± 90°) or *syn* (*χ* = 0 ± 90°) and *θ* (∠(N9–C8–C10–C11)) defines the degree of twist between the nucleobase and the 8-phenyl substituent. (b) Structures of 8arylG probes.

The C-linked 8arylG nucleosides are routinely synthesized from 8-BrG and the appropriate arylboronic acid using the Suzuki–Miyaura cross-coupling reaction.^64^ Calculations (B3LYP/6-31G(d)) reveal that *anti*-structures are less stable than *syn*-structures, mainly since all *syn* minima contain an O5′–H···N3 hydrogen bond (1.80–1.96 Å).^55^,^65^–^67^ The predicted *syn*-preference for the C-linked 8arylG nucleosides is supported by NMR spectra that exhibit a downfield shift of H2′, C1′, C3′ and C4′ and an upfield shift of C2′ compared to the corresponding signals for native G.^67^,^68^ Solid-state structures of C-linked 8arylG nucleosides, including an 8-quinolyl derivative (QG)^24^ and an (*N*,*N*-dimethylaniline)guanosine analog,^69^ are also *syn*. As a specific example, DFT calculations predict the *syn* orientation of the smallest (unsubstituted) 8-phenylG (PhG) derivative (Fig. 3a) to be favoured over *anti* by 25.1 kJ mol^–1^.^24^ In a representative low-energy *syn*-structure, *χ* = 66.8° and the twist angle (*θ*) between the phenyl ring and the nucleobase is 41.2°.^24^ The PhG nucleoside is also fluorescent in H_2_O (*λ*_em_ = 395 nm, *Φ*_fl_ = 0.44 (ref. 24)) following excitation at 277 nm (log *ε* = 4.33 (ref. 70)).

Given that PhG exhibits impressive emission intensity in H_2_O, it became apparent that manipulation of the phenyl ring with various substituents could generate new fluorescent nucleobase analogs with potentially useful emission switching properties (Fig. 3b). For example, the *ortho*-substituted phenolic nucleoside (oPhOHG) can produce a planar conformation in aprotic solvents due to a intramolecular H-bond between the phenolic OH and *N*^7^ of the nucleobase.^71^ The planar species absorbs at ∼320 nm and displays visible emission at 476 nm due to an excited-state intramolecular proton transfer (ESIPT) process to afford the keto tautomer (Fig. 3b).^71^ In H_2_O, the intramolecular H-bond is disrupted and the nucleoside adopts a twisted structure (*λ*_max_ = 280 nm) that displays enol emission at *λ*_em_ = 395 nm (*Φ*_fl_ = 0.44).^71^ This differential fluorescence has proven useful for probing the local solvent environment of oPhOHG within duplex DNA.^72^ The oPhOHG adduct also binds Cu(ii) (log *K*_a_ = 4.59) and Ni(ii) (log *K*_a_ = 3.65) effectively,^73^ and the enol emission is quenched upon such metal coordination, illustrating the ability to use fluorescence to monitor metal ion binding by nucleic acids.^73^ Likewise, the pyridyl derivative (2PyrG) has also been employed for metal ion binding by various DNA topologies.^74^

The attached pyridyl group in 2PyrG extends the Hoogsteen H-bonding face of the G nucleobase by an additional H-bonding acceptor. In contrast, the indole-linked derivative (IndG) extends the Hoogsteen H-bonding face by an H-bonding donor, and exhibits emission at 395 nm (*Φ*_fl_ = 0.78 in H_2_O, *λ*_ex_ = 321 nm) that is sensitive to WC (quenched emission) *versus* Hoogsteen H-bonding (enhanced emission intensity).^75^ The *para*-substituted phenolic nucleoside (pPhOHG) exhibits pH-sensitive fluorescent properties.^76^ The neutral adduct displays emission at 390 nm (*Φ*_fl_ = 0.47 in H_2_O) that is quenched upon phenolate formation (p*K*_a_ ∼ 8.9). This result prompted the synthesis of the 8-(2-chloro-phenol)adenosine analogue (2-Cl-pPhOHA) with a p*K*_a_ of 7.29 for fluorescent pH-sensing activity in the physiological pH range.^76^

The electron-rich nature of the G nucleobase also permits generation of visibly emissive derivatives by attaching electron-withdrawing 8-aryl groups to afford new derivatives with push–pull characteristics.^24^,^42^ The pCNPhG nucleoside displays blue emission at 468 nm that is quenched in H_2_O (*Φ*_fl_ = 0.04), but lights-up in aprotic solvents (*Φ*_fl_ = 0.43 in CH_3_CN).^42^ The 8-quinolyl derivative (QG) displays dual fluorescence in CH_3_CN at 384 and 510 nm. However, since red-shifted twisted intramolecular charge-transfer (TICT) states for QG are strongly quenched in H_2_O, the single peak for QG in H_2_O at 407 nm (*Φ*_fl_ = 0.03) was ascribed to locally excited (LE) emission.^24^ The emission sensitivity to solvent polarity displayed by these donor–acceptor adducts has proven useful for predicting adduct conformation in duplex structures.^24^

**Scheme 1:**
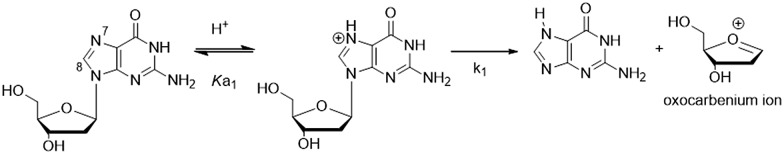
Acid-catalyzed hydrolysis of 2′-deoxyguanosine.

Modification of the 8-site of G with aryl groups also accelerates the rate of acid-catalyzed hydrolysis.^70^ Hydrolysis of the unmodified base (Scheme 1) proceeds *via* a stepwise mechanism, involving initial protonation at *N*^7^ (p*K*_a_ of the protonated species in 2.34 (ref. 77)) followed by rate-limiting unimolecular cleavage of the glycosidic bond (*k*_1_).^22^,^70^ The oxocarbenium ion subsequently undergoes hydration to produce the 1′-hydroxylated-2′-deoxyribose sugar. Rates of hydrolysis for 8arylG adducts are 5- to 45-fold greater than the unmodified base in 0.1 M HCl, which increases to 9- to 200-fold at pH 4.^70^ The relative depurination efficiencies of C-linked 8arylG adducts can be rationalized by the relative relief of steric strain in the twisted nucleoside (about the base–aryl moiety bond) upon deglycosylation, which yields a less twisted or even planar nucleobase.^70^ Thus, the presence and chemical composition of the bulky moiety in C-linked 8arylG lesions can have a significant effect on the structure and properties of the isolated nucleoside.

## Incorporation into oligonucleotides

**Fig. 4:**
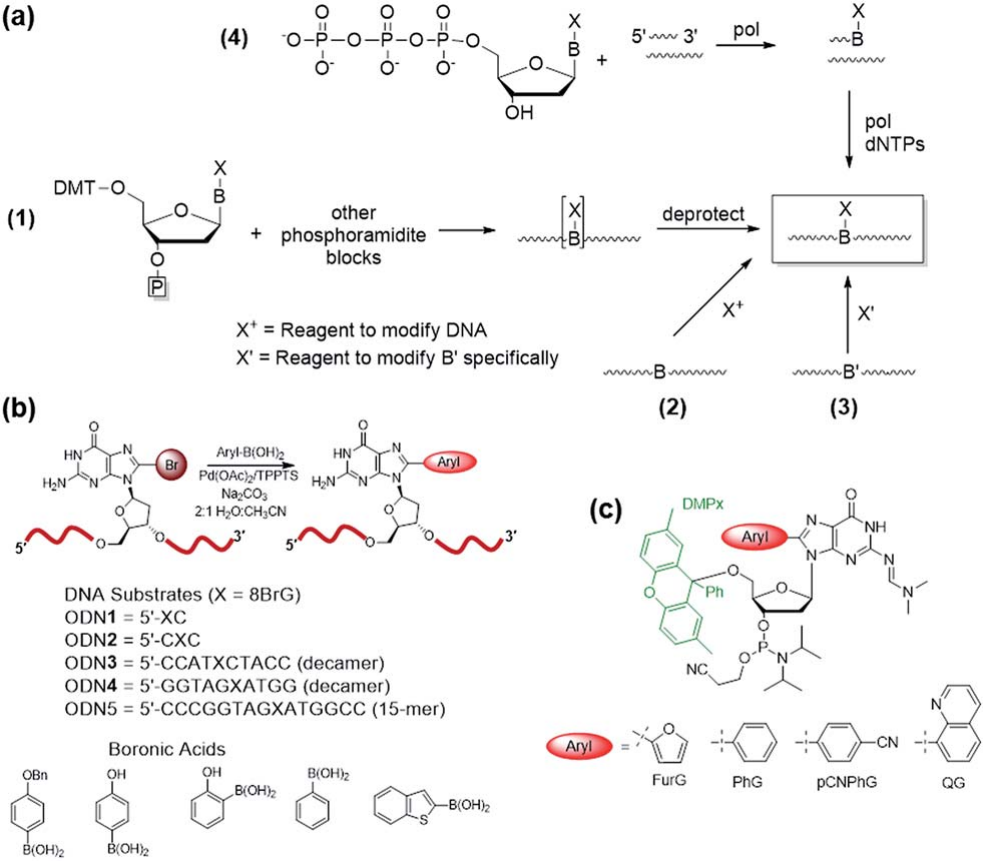
(a) Methods for synthesis of oligonucleotides containing modified DNA bases at defined positions. (b) Postsynthetic Suzuki–Miyaura cross-coupling of 8BrG-modified oligonucleotides for synthesis of 8arylG-modified DNA substrates. (c) Utility of the DMPx group for solid-phase synthesis of oligonucleotides containing acid-sensitive 8arylG adducts.

Four common approaches are utilized to incorporate modified DNA bases into oligonucleotides in a site-specific fashion (Fig. 4a).^78^ The phosphoramidite approach (Fig. 4a(1)) requires the chemical synthesis of the modified phosphoramidite that can be incorporated into oligonucleotides using a DNA synthesizer. The modified phosphoramidite must be compatible with the DNA synthesis conditions (acidic, alkaline and oxidative) and deprotection (strongly alkaline) of the DNA strand.^1^ Alternatively, postsynthetic strategies may involve treating an oligonucleotide with a reagent that will modify the desired nucleobase to generate the desired lesion (Fig. 4a(2)). An inherent problem with this approach is that multiple modifications may occur and positional isomers can be difficult to separate. To circumvent some of these challenges, an appropriately modified base may be incorporated site-specifically into an oligonucleotide and then chemically modified to generate the desired final product (Fig. 4a(3)). Finally, modified deoxynucleotide triphosphates may be incorporated enzymatically into a primer strand by the action of a DNA polymerase (Fig. 4a(4)).^79^ Nevertheless, this approach is limited by the activity of polymerases toward a variety of bulky damaged products.

For site-specific incorporation of 8arylG bases into oligonucleotide substrates, we were concerned that their sensitivity to acids and oxidants may pose issues for their synthesis using the solid-phase approach with 8arylG phosphoramidites. The Hocek laboratory also reported that 8-Ph-dATP is too bulky to be a polymerase substrate,^79^ which renders an enzymatic approach unfeasible. Thus, we developed a postsynthetic strategy utilizing the palladium-catalyzed (Suzuki–Miyaura) cross-coupling reaction (Fig. 4b).^80^ In this strategy, the commercially available phosphoramidite of 8BrG is incorporated site-specifically into various oligonucleotide substrates using solid-phase DNA synthesis and then reacted with a range of arylboronic acids. This strategy avoids exposure of the 8arylG base to acids and oxidants, and can be employed to incorporate a single adduct into relatively short DNA substrates (3–15mers). However, the strategy has a number of limitations, including poor yields, the inability to incorporate multiple adducts into strands and limitations in generating the longer adducted DNA templates that are required to assess the biological impact of the lesion using DNA polymerases or repair enzymes.

A more efficient protocol for synthesis of DNA substrates containing 8arylG bases utilizes the 5′-*O*-2,7-dimethylpixyl (DMPx) protecting group (Fig. 4c) in a solid-phase assisted synthesis strategy.^81^ The DMPx group is more acid-labile than DMT (release of an aromatic carbocation *versus* a benzylic carbocation) and can be efficiently removed using 0.5% dichloroacetic acid (DCA) in dichloromethane rather than the 3% DCA required to remove the 5′-*O*-DMT protecting group.^82^

Indeed, we successfully employed the DMPx group to incorporate a range of 8arylG bases (FurG, PhG, pCNPhG, QG, Fig. 4c) into DNA substrates using solid-phase synthesis. For incorporation of FurG, which exhibits a 55.2-fold increase in hydrolysis rate compared to dG in 0.1 M aqueous HCl, the 5′-*O*-DMPx group provided a 4-fold yield increase compared to use of the 5′-*O*-DMT group.^81^ However, attempts to purify the modified DNA substrates with the final 5′-*O*-DMPx group attached using commercially available solid-phase extraction cartridges resulted in degradation of the oligonucleotide through exposure of the 8arylG base to both acid and water.^81^ Therefore, although 8arylG bases can be incorporated into DNA substrates using solid-phase synthesis (DMPx or DMT protection), it is critical to remove the final 5′-OH protecting group on-column prior to cleavage of the oligonucleotide from the solid support using aqueous ammonium hydroxide.

## Structural and biological impact

**Fig. 5:**
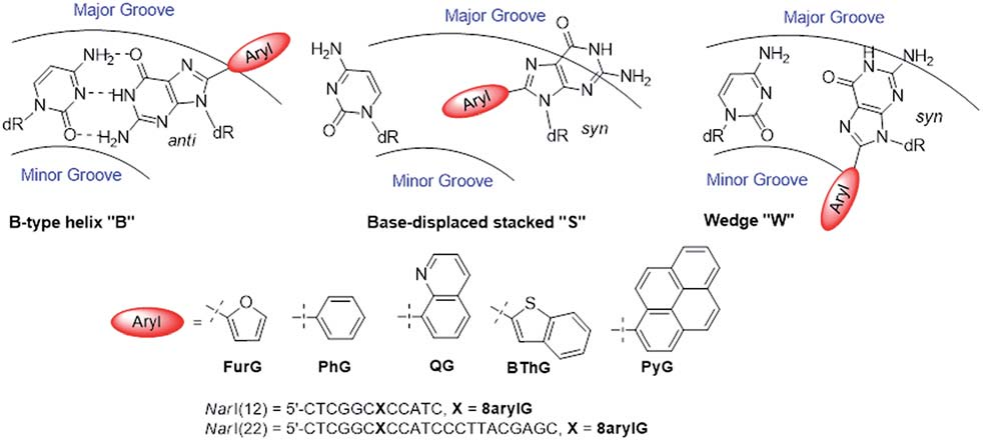
Depictions of the three major conformations produced by 8arylG adducts, various 8aryl groups used to model C-linked 8arylG adducts and oligonucleotide sequences of *Nar*I(12) and *Nar*I(22).

As a first step toward understanding the biological impact of bulky DNA adducts, efforts have been made to relate adducted duplex structures to mutagenic outcomes. For N-linked 8arylG adducts, three distinct conformational themes have been characterized for the associated adducted DNA by NMR spectroscopy, which depend on the (*anti* or *syn*) glycosidic bond orientation and the location of the 8aryl group within the duplex (Fig. 5).^29^,^34^ In the B-type conformation, the adduct adopts the *anti*-glycosidic orientation and maintains WC H-bonding with the opposing base C, which positions the 8aryl group in the solvent-exposed major groove. In the base-displaced stacked (S-type) conformation, the adduct adopts the *syn*-orientation and stacks the 8aryl ring between the flanking base pairs, which displaces the opposing C out of the helix. In the wedge (W-type) conformation, the 8arylG adduct also adopts the *syn*-conformation, but the 8aryl moiety is positioned in the minor groove and the opposing base remains in the helix. The distribution amongst the various conformations is dependent on the nature of the attached 8aryl moiety (planar-fused multiring systems *versus* single ring or nonplanar groups) and the neighbouring base sequence context.^29^,^34^ For example, the N-linked 8arylG adduct produced by AAF induces all three conformational themes in normally paired DNA duplexes, while changes in the linker substitution results in the B and S-type conformations for the N-linked adduct produced by AF.^29^,^32^–^34^ Thus, each adduct yields multiple conformations of adducted DNA, with each conformation leading to different mutational outcomes. In general, the B-type conformation will favour insertion of the correct base C opposite the lesion, but further extension can be both error free and error prone.^32^ The mutagenic arrangement following C insertion opposite the N-linked adduct is induced by interactions between the polymerase and the bulky fluorenyl rings rather than by a base pair-stabilized misalignment.^32^ The S-type conformation is strongly blocking because the fluorenyl rings occupy the position of the incoming dNTP within the polymerase; the S-type conformation is also associated with the production of deletion mutations.^29^ The W-type conformation is strongly associated with misincorporation due to hydrogen bonding interactions between the incoming mismatched dNTPs with the Hoogsteen-face of the *syn*-adduct.^29^

C-Linked 8arylG adducts are much more rigid than the N-linked counterparts because they lack a flexible tether separating the 8aryl ring from the G nucleobase. They also tend to be highly twisted about the nucleobase–aryl moiety bond in order to reduce steric interactions that arise due to the closer proximity of the aryl group and sugar moiety, as well as decreased inherent flexibility within the bulky moiety, in the absence of the tether. As a result, C-linked 8arylG adducts likely exhibit a decreased tendency to produce the highly blocking/mutagenic S-type conformation compared to their N-linked counterparts. Nevertheless, the formation of C-linked 8arylG adducts has also been implicated in a variety of mutagenic outcomes. For example, arylhydrazines, which generate phenyl radicals, produce 8PhG adducts that are mutagenic in bacteria.^60^–^62^ Benzo[*a*]pyrene (B[*a*]P) undergoes peroxidase-mediated oxidation to afford radical cations, where the potential involvement of an 8B[*a*]PG adduct produces transversion (G → T and G → C) mutations in yeast.^83^ The phenolic mycotoxin ochratoxin A (OTA) produces a bulky C-linked 8arylG adduct,^35^–^37^ and increases the mutational frequency, as well as the induction of deletion mutations and double strand breaks, in the kidneys of male rats.^84^

**Fig. 6:**
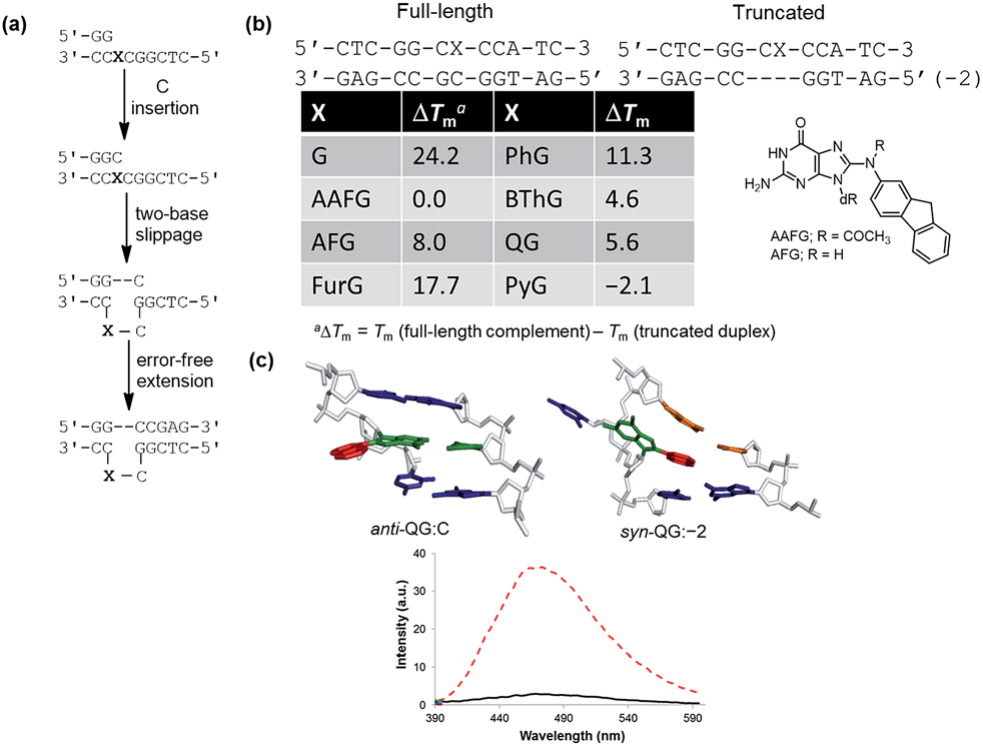
(a) Proposed model for two-base slippage induced by N-linked 8arylG lesions in a CpG dinucleotide repeat sequence. (b) Thermal melting (*T*_m_) values for 8arylG lesions in full-length relative to truncated *Nar*I(12) duplexes. (c) Most stable MD structures of QG (quinolyl group in red, G nucleobase in green) in *Nar*I(12) paired opposite C (*anti*-QG:C), within the truncated duplex (*syn*-QG:–2) and emission spectra (*λ*_ex_ = 330 nm) of QG within the full-length (solid black trace) and truncated (dashed red trace) duplexes.

To demonstrate relationships between 8aryl ring size, adduct conformation and *in vitro* mutagenicity for C-linked 8arylG adducts, five adducts with differing ring types (FurG, PhG, QG, BThG and PyG) were incorporated into the reiterated G_3_-position (X) of the *Nar*I type II restriction endonuclease recognition sequence (*i.e. Nar*I(12) and *Nar*I(22), Fig. 5).^24^,^28^ The G_3_-site of *Nar*I is part of a CpG dinucleotide repeat that is a hotspot for two-base deletion mutations induced by polycyclic N-linked 8arylG adducts in bacteria *via* a two-base slippage mechanism (Fig. 6a).^85^ Optical experiments (ultraviolet (UV) thermal melting, circular dichroism (CD) and fluorescence) combined with molecular dynamics (MD) simulations were utilized to define the structural features of the adducted *Nar*I(12) duplexes.^24^,^28^ The adducted *Nar*I(22) templates were also employed in primer elongation experiments to assess adduct impact on DNA replication *in vitro* by the polymerases-*Escherichia coli* DNA polymerase I Klenow fragment exo^–^ (Kf^–^), and DNA polymerase IV from *Sulfolobus solfataricus* P2 (Dpo4). High-fidelity polymerases, such as Kf^–^, can fit one templating nucleotide in their active site, and favour accurate replication when correct WC base pairing is established with the incoming deoxynucleotide triphosphate (dNTP).^86^ Bulky DNA adducts often stall or block DNA replication by high-fidelity polymerases, which *in vivo* is believed to be a signal for the recruitment of Y-family translesion polymerases. The Y-family polymerases have spacious solvent-exposed active sites that can accommodate bulky DNA lesions, while facilitating low-fidelity DNA replication.^78^,^86^ Dpo4 is regarded as a prototypical Y-family polymerase that serves as an excellent model for investigating how structural features of adducts determine lesion bypass efficiency and fidelity.

In the *Nar*I(12) duplex, C-linked 8arylG adducts paired opposite the correct base C strongly decrease duplex stability compared to the unmodified control due to their energetic preference for the *syn*-conformation.^24^,^28^ More importantly, the ability of a lesion to stabilize the slippage product relative to the full-length duplex correlates with an ability to induce –2 frameshift mutagenesis in bacteria. As a result, at the G_3_-position of *Nar*I, thermal melting parameters (*T*_m_ values) of the full-length complement duplex (with the adduct paired opposite the correct base C) have been compared to *T*_m_ values of *Nar*I(12) hybridized to a truncated 10mer (two-base deletion) sequence (–2) (Fig. 6b).^87^ The full-length complement and truncated duplexes containing the N-linked 8arylG adduct of AAF (AAFG) have the same *T*_m_ (Δ*T*_m_ = 0.0 °C, Fig. 6b). This highlights the ability of the AAFG lesion to stabilize the slippage product,^87^ which correlates with reports that AAFG produces –2 deletions (91% mutational frequency) at the G_3_-position in *Nar*I in bacteria.^88^ In contrast, the AFG lesion stabilizes the slippage product to a lesser degree (Δ*T*_m_ = 8.0 °C)^87^ and leads to only base-pair substitution mutations.^88^ For the C-linked 8arylG adducts in *Nar*I(12), full-length duplexes containing the single-ringed derivatives (FurG and PhG) are significantly more stable than the truncated duplex, suggesting an inability of these lesions to induce –2 deletions by stabilizing the slippage product. In contrast, the larger fused-ring derivatives (BThG, QG and PyG) exhibit similar *T*_m_ values for the full-length and truncated duplexes, with the truncated duplex being more stable for the bulkiest PyG adduct (Δ*T*_m_ = –2.1 °C). These findings suggest that these lesions may induce deletion mutations when inserted into repeat sequences that are prone to slippage.^24^,^28^

MD simulations were carried out on the full-length and truncated duplexes, and adduct conformational preferences were ranked according to the calculated free energies.^24^,^28^ In the full-length duplex with the lesion paired opposite C, C-linked 8arylG adducts favour the major groove (B-type, Fig. 5) conformation, although alternative *syn*-conformations are energetically accessible for FurG, PhG, BThG, QG (all W-type) and PyG (S-type). In the truncated duplexes, FurG, PhG and BThG favour the *anti*-conformation, while QG and PyG favour the *syn*-conformation. For the push–pull QG, its emissive response was consistent with the preferred conformation predicted by the MD simulations (Fig. 6c). Specifically, in the full-length duplex, the *anti*-conformation of QG is favoured over *syn*-structures by at least 25 kJ mol^–1^, which preferentially exposes the quinolyl moiety to the bulk aqueous solvent in the major groove (*anti*-QG:C, Fig. 6c), and quenches CT emission of QG (solid black emission trace, Fig. 6c). In contrast, the *syn*-conformation of QG in the truncated duplex sequesters the quinolyl moiety from the bulk aqueous solvent (*syn*-QG:–2, Fig. 6c) and results in a 12-fold increase in CT emission intensity (dashed red emission trace, Fig. 6c).^24^

In primer elongation assays using the *Nar*I(22) templates, C-linked 8arylG adducts strongly block DNA replication by the high-fidelity DNA polymerase Kf^–^ following insertion of a single base opposite the lesion, typically the correct base C.^24^,^28^ This observation was consistent with their preference for the B-type structure, as predicted by MD simulations. In single-nucleotide incorporation assays, the smallest lesion FurG produced the greatest levels of misincorporation (A > G ≫ T), which correlations with this lesion resulting in the smallest energy difference between the B and W-type adducted DNA conformations.^24^ Using Dpo4 as a model translesion polymerase, C-linked 8arylG adducts were found to cause targeted (at the lesion site, *i.e.* base substitution) and semi-targeted (in the vicinity of the lesion site, *i.e.* deletion) mutations.^24^,^28^ The single-ringed derivatives (FurG and PhG) produced the greatest levels of misincorporation (A and G),^24^ while the fused-ringed derivatives (QG, BThG and PyG) strongly blocked extension by Dpo4.^24^,^28^

**Fig. 7:**
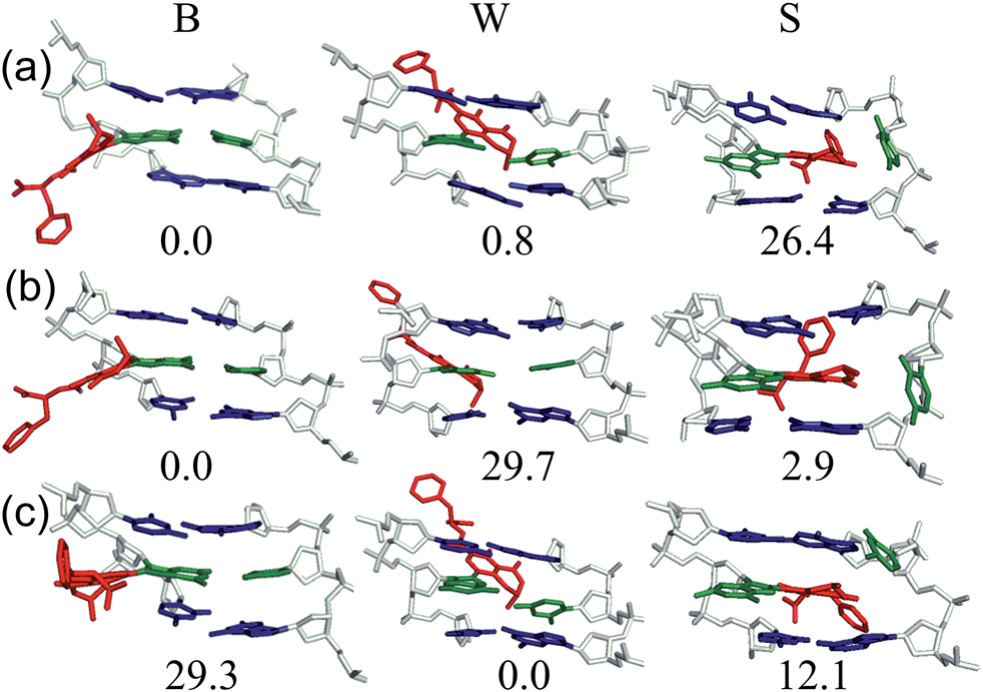
Representative MD structures and relative stability (Δ*G*, kJ mol^–1^) of the B (left), W (middle) and S (right) conformers of the monoanionic OTAG adduct incorporated into the (a) G_1_, (b) G_2_ or (c) G_3_ position in the *Nar*I(12) duplex.

Despite the reported reduced flexibility of the C-linked adducts discussed thus far, adducted DNA associated with the fungal carcinogen OTA has a much more complicated conformational profile. Although the genotoxicity of OTA has been debated in the literature, several studies have illustrated that OTA primarily results in a C-linked 8arylG adduct.^35^,^36^ Using MD simulations, a dynamic conformational profile has been revealed for the C-linked OTAG lesion that involves at least two of the B, W and S-type conformations, with the energetically accessible orientations being highly dependent on the lesion site sequence context (Fig. 7), as well as the OTA ionization state.^37^ This mixture of conformations correlates with the complicated mutagenic profile associated with OTA exposure, which includes base deletions and double strand breaks.^84^ Furthermore, the adoption of the S-type conformation according to MD, coupled with observed double strand breaks, suggests that this bulkier C-linked adduct may inhibit replication.^37^ Nevertheless, the genotoxic effects of OTA have been reported in only select tissues (outer stripe of the outer medulla rather than the entire kidney^84^), which suggests that certain conformations may dominate in particular cellular environments. This example highlights the complex interplay that can exist between the conformations adopted and biological outcomes for C-linked 8arylG adducts.

Alternative mechanisms for the toxicity of 8arylG adducts may stem from their ability to induce alternative DNA conformations. For example, the Gannett laboratory has examined the impact of 8PhG lesions on Z-DNA formation.^60^–^62^ Formation of Z-DNA *in vivo* has been implicated in mutagenesis by stimulating large-scale gene deletions, translocations, and rearrangements.^57^ A major structural requirement for Z-DNA formation is sequence (alternating purine–pyrimidine bases) and the addition of cationic species to screen electrostatic repulsion between adjacent phosphate groups in Z-DNA. It is also known that methylation of cytosine at the 5-carbon position in CpG runs can favour Z-DNA formation.^89^ In an initial effort to gauge the impact of an 8PhG adduct on the B-/Z-DNA equilibrium, the simplest member 8PhG along with four *para*-substituted 8RPhG derivatives (R = CH_3_, CH_2_OCH_3_, CH_2_OH and CO_2_^–^) were incorporated into d(CGCGCXCGCG)_2_ (X = 8PhG or 8RPhG).^60^,^61^ The effect of 8PhG on the B/Z equilibrium was determined using CD by monitoring the salt concentration required to generate a B/Z equilibrium equal to 1. The unmodified duplex required a salt concentration (NaCl) of ∼3.2 M, while introduction of the 8PhG lesion lowered the salt concentration by a factor of ∼3–25, depending on the nature of the *para*-substituent. These results suggest that 8PhG adducts change the position of the B/Z equilibrium by destabilizing the B-form rather than stabilizing the Z-form.^60^,^61^

Unfortunately, the duplex model previously used to determine the B/Z equilibrium was not ideal because it contained two 8PhG modifications that could potentially provide an additive or synergistic effect to favour the Z-form.^62^ Furthermore, it would be highly unlikely that two 8PhG adducts would form on consecutive base steps *in vivo*. Thus, a hairpin sequence (d-(CG)_5_T_4_(CG)_5_) containing an intramolecular (CG)_5_ duplex was synthesized in order to incorporate only one 8PhG modification.^62^ Under physiological conditions (2 mM MgCl_2_, 10 mM NaCl, 140 mM KCl and 1 mM spermine, 37 °C, pH 7.4) the hairpin preferentially adopted the Z-form structure. This result strengthened the argument that 8PhG lesions favour Z-DNA through destabilization of the B-form, which may have biological significance. Specifically, sequences that can produce Z-DNA are most commonly found in gene promoter regions where Z-DNA can regulate transcription and nucleosome positioning.^57^ Therefore, carcinogens that produce 8arylG adducts may overwhelm the normal cellular response to Z-DNA formation and lead to large-scale deletions in mammalian cells.^62^

**Fig. 8:**
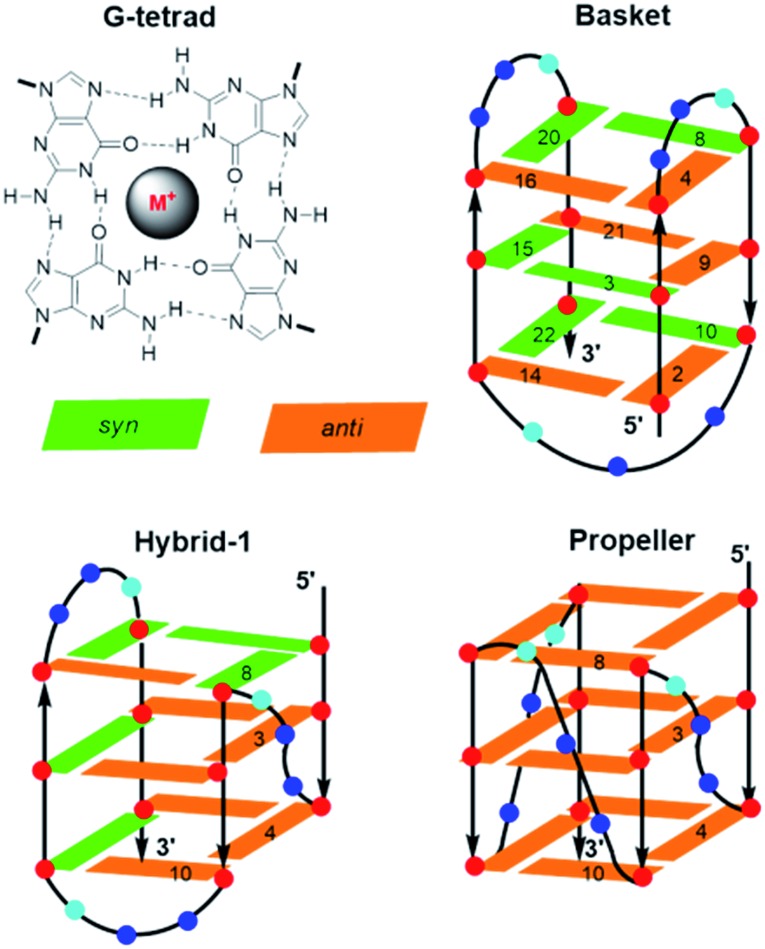
Structures of a G-tetrad and the basket, hybrid and propeller folds of GQs produced by HTelo22. The 8arylG probe FurG was inserted into positions 3, 4, 8 or 10 of the GQs, *anti*-Gs are shown in brown, *syn*-Gs are shown in green.

Other unique DNA topologies that are more frequent in gene promoter regions include GQs.^59^ Chromosome ends (telomeres) are capped with kilobase-long runs of the repeating 5′-(TTAGG)_*n*_-3′ sequence.^90^ Although most of the telomere resides as a duplex, the 3′-terminal 50–200 nucleotides are single-stranded and can fold into various GQ topologies. As described earlier, GQs are assembled through the sequential stacking of G-tetrads around a metal cation (Fig. 8), with the intervening sequences extruded as single-strand loops. The GQs topologies are classified depending on the orientation of the DNA strands. They can be parallel (all Gs in the tetrad are in the *anti*-conformation), antiparallel (alternating *syn*- and *anti*-Gs) or hybrids thereof. Model studies utilizing a four-repeat section of the human telomeric DNA sequence (HTelo22, (d[AG_3_(T_2_AG_3_)_3_])) have demonstrated extensive GQ polymorphism (Fig. 8).^63^ In Na^+^ solution, HTelo22 produces a basket-type antiparallel GQ,^58^ while in K^+^ solution, mixed parallel/antiparallel (hybrid-1 or hybrid-2) GQ structures are the major conformations, with hybrid-1 being the major fold (Fig. 8).^91^ In the crystalline state^92^ containing K^+^ or in K^+^ solution containing certain additives (*i.e.*, CH_3_CN, ethanol and polyethylene glycol (PEG)),^93^ a propeller-type parallel-stranded GQ structure is favoured. Topologies of HTelo22 formed in K^+^ (hybrid or parallel) are expected to be more biologically relevant than the antiparallel structure produced in Na^+^, because GQs have a stronger binding constant with K^+^, which is present in a higher cellular concentration (∼140 mM [K^+^] *versus* ∼10 mM [Na^+^]).^94^

The G-rich nature of the human telomere sequence makes it highly susceptible to electrophilic attack.^94^ Hence, model studies have been conducted to determine the impact of the 8oxoG lesion on GQ formation by HTelo.^94^,^95^ The lesion strongly perturbs GQ stability due to the inability of 8oxoG to form Hoogsteen base pairs with G. Placement of 8oxoG in an exterior tetrad forces an antiparallel topology in K^+^ solution, while an unstable triplex-like topology is produced with 8oxoG in the middle tetrad.^96^ Such studies prompted our laboratory to examine the structural impact of an 8arylG adduct on the GQ polymorphism exhibited by HTelo22.^63^ Unlike 8oxoG, 8arylG adducts can form Hoogsteen base pairs with G; however, their strong *syn*-preference may perturb or inhibit formation of certain GQ topologies that could impact telomeric function. As a representative 8arylG lesion, FurG was incorporated into various G-tetrad positions (3, 4, 8 or 10, see Fig. 8 for numbering) of HTelo22.^63^ On the basis of CD signatures, *T*_m_ analysis and fluorescence measurements, all FurG-modified HTelo22 sequences adopt the antiparallel fold in Na^+^ solution, with *T*_m_ values higher than the native HTelo22. However, in K^+^ solution, sequences with FurG at positions 3 and 4 exclusively form the antiparallel basket GQ that is produced in Na^+^ solution, while FurG at positions 8 and 10 produces the expected hybrid GQ that is favoured by the native HTelo22 sequence. At all positions examined, the FurG modification strongly impedes formation of the parallel fold that is produced by the native HTelo22 sequence in K^+^ solution in the presence of certain additives. These results demonstrate that production of 8arylG adducts within the human telomeric sequence may make it difficult to form certain GQ topologies, which could impact chromosome stability. Nevertheless, 8arylG adducts can stabilize GQ structures when placed in *syn*-G sites. The ability to stabilize GQ structures has important biological implications. For example, GQ formation can induce replication dependent double strand breaks.^97^ Together, the above studies highlight the diverse structural and biological impacts of C-linked 8arylG adducts when incorporated into DNA structures.

## Utility as fluorescent probes

Aptamers are single-stranded DNA, RNA or modified nucleic acids that are designed to fold into unique three-dimensional shapes, which are then recognized by a target molecule.^98^,^99^ Given the stability and diversity of GQs, it is not surprising that many aptamers have been designed based on the GQ structural motif.^100^ The antiparallel GQ scaffold provides an opportunity to employ fluorescent 8arylG probes for GQ detection strategies. Compared to other fluorescent guanine analogues, such as pteridines (3-MI and 6-MI)^9^ and benzo-expanded guanine derivatives,^11^ 8arylG probes are relatively easy to synthesize and have a *syn*-preference making them useful for defining the G conformation within the tetrad; the probe will generally stabilize the GQ when placed in a *syn*-G position, but destabilize the GQ when placed in an *anti*-G position. Furthermore, the G–G base-stacking, hydrogen bonding, and restricted motions within GQ structures can enhance the photoexcited lifetimes and energy transfer properties of a G residue.^38^ These factors can amplify the emission of the 8arylG probe within the GQ structure compared to its emission in the duplex. Thus, 8arylG probes can be effective diagnostic tools in duplex–GQ exchange systems that are commonly employed in DNA-based detection strategies.^42^,^52^,^53^ The level of precision offered by internal fluorescent nucleobase mimics can complement other approaches including end-labelled dyes and label-free dyes, because the internal probes may assist in establishing Gs in the tetrad *versus* loop positions, site(s) of ligand binding, and remove false negatives or positives that can occur with the use of external dyes that are not covalently attached to the aptamer.^101^

**Fig. 9:**
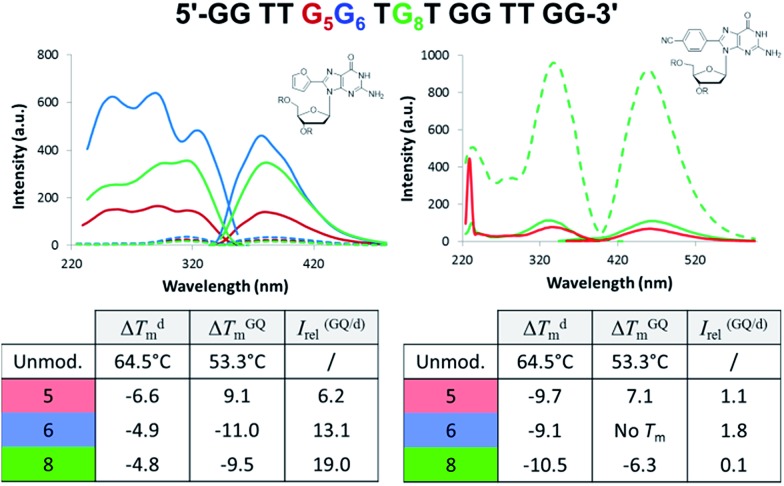
TBA sequence with G_5_, G_6_ and G_8_ highlighted in red, blue and green, respectively. Excitation and emission spectra for mTBA with duplexes as dashed traces, GQs solid traces and different colours for the various positions of the 8arylG probe within mTBA. Thermal melting parameters for duplex (d) and GQ in K^+^ solution for mTBA compared to the unmodified sequence.

To test 8arylG probe performance in an antiparallel GQ aptamer, FurG and the donor–acceptor pCNPhG were inserted into various positions within the thrombin binding aptamer (TBA, Fig. 9).^42^ The two 8arylG probes were inserted into the 5, 6 or 8 position that represent a *syn*-G position within a G-tetrad (G_5_), an *anti*-G position (G_6_) or a diagonal TGT loop position (G_8_). The duplex and GQ structures produced by the modified-TBA (mTBA) strands were compared to the corresponding structures adopted by native TBA using CD, *T*_m_ analysis, fluorescence and MD simulations.^42^

In the duplex structure, the emission of the FurG probe (*λ*_em_ = 384 nm, *Φ*_fl_ = 0.49 (ref. 67)) was strongly quenched (dashed traces, Fig. 9), while the emission exhibited a 6- to 19-fold increase in fluorescence intensity in the chair-like antiparallel GQ (table, Fig. 9) compared to the duplex emission. The excitation spectra for FurG in the GQ also displays diagnostic energy-transfer bands at ∼255 and 290 nm that are absent in the duplex excitation spectra. The probe had a destabilizing influence on duplex stability (Δ*T*_m_ ∼ –5 °C) and on GQ stability when placed at *anti*-G_6_ and in the diagonal loop G_8_. However, at the *syn*-G_5_ position, FurG increases GQ stability by ∼9 °C. The bulkier pCNPhG probe had a stronger destabilizing influence on duplex stability and inhibited GQ formation when placed in the *anti*-G_6_ position (*T*_m_ value could not be determined). Interestingly, the probe was not as destabilizing as FurG in the GQ when placed in the diagonal G8 loop position (due to increased stacking with the G-tetrad); the pCNPhG probe also stabilized the GQ at *syn*-G_5_ (by ∼7 °C). In contrast to the FurG probe, the push–pull pCNPhG exhibited quenched emission in the GQ structures at positions G_5_ and G_8_, and at position G_5_ in the duplex. However, in the duplex at position G_8_, where the probe is flanked by T residues, the probe was strongly emissive (∼10-fold increase compared to the GQ structure). Thus, FurG serves as an effective turn-on emissive probe in duplex–GQ exchange, while pCNPhG can be employed as a turn-off probe when inserted into the diagonal G_8_ loop position of TBA.^42^ Indeed, duplex–GQ exchange studies with FurG at *syn*-G_5_ and pCNPhG at G_8_ within mTBA have demonstrated the utility of these probes for K^+^ ion^42^ and thrombin detection.^52^

**Fig. 10:**
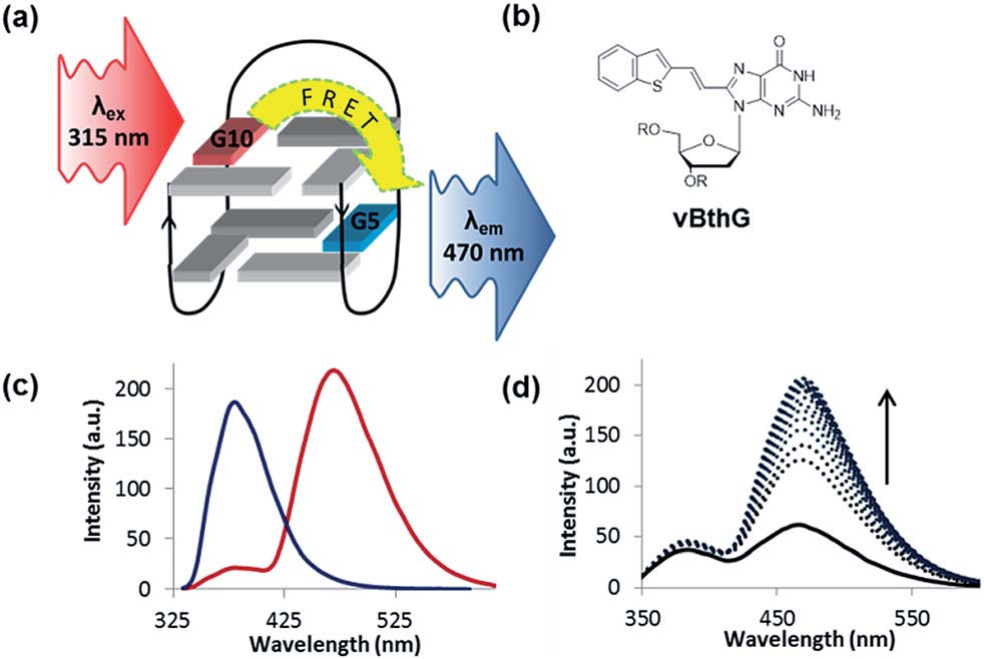
(a) Schematic for FRET in TBA using FurG as the donor (D, *λ*_ex_ = 315 nm) paired with an acceptor (A, *λ*_em_ = 470 nm) in the G-tetrad. (b) Structure of the A probe vBthG. (c) Emission spectra (*λ*_ex_ = 315 nm) of mTBA in K^+^-solution with FurG (D, *syn*-G_10_) in the absence (blue trace) and presence (red trace) of vBthG (A, *syn*-G_5_) for a FRET efficiency of 88% in the antiparallel GQ. (d) Thrombin emission titration with mTBA (D/A, 10; 5) with *λ*_ex_ = 315 nm.

The turn-on emissive properties of FurG upon GQ folding suggested that it could serve as a donor (D) dye to be paired with an acceptor (A) G derivative for diagnostic fluorescence resonance energy transfer (FRET) signalling for GQ formation (Fig. 10a).^53^ This prompted the synthesis of 8-vinyl-benzo[*b*]thienyl-dG (vBthG, Fig. 10b), which has an absorbance maxima at ∼380 nm and will yield effective spectral overlap with the emission of FurG. The vBthG probe provides visible blue emission at 473 nm (*Φ*_fl_ = 0.29), but is not effective by itself for monitoring duplex–GQ exchange because its emission lacks sensitivity to the change in DNA topology.^53^,^101^ However, when paired with FurG within *syn*-G positions of mTBA (*i.e.* D; A, 10; 5), the D base can act as a switch upon GQ formation, turning on the visible fluorescence from the vBthG probe. The FRET efficiency of the D/A pair in the antiparallel GQ was 88% at positions 10; 5 (Fig. 10c). The mTBA sample with the probes at *syn*-G_5_ (A) and *syn*-G_10_ (D) strongly decreased duplex stability (by 20 °C), but increased GQ stability (by ∼9 °C) and acted as an effective turn-on duplex–GQ exchange system (4-fold increase in emission intensity at ∼470 nm upon thrombin binding, Fig. 10d).^53^ Overall, the ability of 8arylG bases to exhibit emission switching properties upon change in DNA topology (duplex–GQ exchange) provides a basis for their utility in DNA-based diagnostics.

## Conclusions and future prospects

The literature summarized above highlight the structural and *in vitro* mutagenic impact of C-linked 8arylG lesions together with their potential applications as fluorescent probes in duplex–GQ exchange systems. In terms of biological impact, until recently, few studies have focused on the C-linked 8arylG variety despite the fact that many chemical mutagens are known to produce such adducts. Our studies to date demonstrate that C-linked 8arylG lesions are stronger blocks of DNA synthesis than the corresponding N-linked derivatives, and can promote polymerase slippage and misincorporation. Future efforts should examine the conformational preferences of C-linked 8arylG adducts in both duplex structures and in template:primers bound to DNA polymerases using high-resolution structural studies combined with MD simulations. In order for C-linked 8arylG adducts to induce mutagenicity, they must evade repair by nucleotide excision repair (NER) enzymes. NER of C-linked 8arylG adducts has yet to be examined. Here, it may be possible to take advantage of the fluorescent nature of C-linked 8arylG adducts to determine incision rates mediated by NER. Fluorescence-based assays for nucleotide selection preferences of Y-family polymerases opposite C-linked 8arylG adducts may also be highly insightful, especially in cases where the lesion induces –2 base slippage; C-linked 8arylG adducts with push–pull character can exhibit enhanced emission when sequestered from the aqueous solvent (*i.e.* within a 2-base bulge). Finally, C-linked 8arylG adducts with defined structures have yet to be incorporated into DNA vectors for the determination of mutational frequency in cell-based assays. Such studies would better permit direct comparisons between the mutagenicity of C-linked and N-linked 8arylG lesions so that the biological impact of linkage type can be established.

In terms of the utility of fluorescent 8arylG probes in duplex–GQ exchange systems, the 8-furyl derivative FurG exhibits favourable turn-on emission switching properties upon change in DNA topology due to energy-transfer from the unmodified Gs in the tetrad that is absent in the duplex structure. Specifically, the base can serve as a donor probe to be paired with an acceptor G for effective FRET signalling of GQ formation. So far, our studies have been limited to TBA, which forms an antiparallel GQ and can be used as “proof-of-principle” to test probe performance. However, many aptamers produce GQs upon target binding, including those for OTA, nucleolin, insulin, HIV-1 reverse transcriptase and ATP.^100^ Many C-rich aptamers also exist that can be paired with GQ-producing complementary strands, such as aptamers for microcystins.^102^ In this scenario, target binding to the aptamer releases the complementary strand containing the 8arylG probe, which is free to fold into a GQ to signal target binding. We expect 8arylG bases to have commercial applications in aptasensors over the next few years. One goal of our laboratories is to develop 8arylG probes that undergo excitation with visible light and can be readily employed at *anti*-G positions for applications within parallel GQ structures. This issue is particularly challenging because low energy emission is usually associated with large chromophores that may inhibit GQ folding or produce CT states that exhibit quenched emission in H_2_O or a lack of emission sensitivity to changes in DNA topology. Nevertheless, the successes registered to date regarding the range of properties and applications of 8arylG bases help ensure that achieving these ambitious goals will push the current boundaries in many nucleic acid-based technologies.
